# Fair regression for health care spending

**DOI:** 10.1111/biom.13206

**Published:** 2020-01-06

**Authors:** Anna Zink, Sherri Rose

**Affiliations:** ^1^ PhD Program in Health Policy Harvard University Cambridge Massachusetts; ^2^ Department of Health Care Policy Harvard Medical School Boston Massachusetts

**Keywords:** constrained regression, fairness, penalized regression, risk adjustment

## Abstract

The distribution of health care payments to insurance plans has substantial consequences for social policy. Risk adjustment formulas predict spending in health insurance markets in order to provide fair benefits and health care coverage for all enrollees, regardless of their health status. Unfortunately, current risk adjustment formulas are known to underpredict spending for specific groups of enrollees leading to undercompensated payments to health insurers. This incentivizes insurers to design their plans such that individuals in undercompensated groups will be less likely to enroll, impacting access to health care for these groups. To improve risk adjustment formulas for undercompensated groups, we expand on concepts from the statistics, computer science, and health economics literature to develop new fair regression methods for continuous outcomes by building fairness considerations directly into the objective function. We additionally propose a novel measure of fairness while asserting that a suite of metrics is necessary in order to evaluate risk adjustment formulas more fully. Our data application using the IBM MarketScan Research Databases and simulation studies demonstrates that these new fair regression methods may lead to massive improvements in group fairness (eg, 98%) with only small reductions in overall fit (eg, 4%).

## 1 INTRODUCTION

Risk adjustment is a method for correcting payments to health insurers such that they reflect the cost of their enrollees relative to enrollee health. It is implemented by most federally regulated health insurance markets in the United States, including Medicare Advantage and the individual health insurance Marketplaces created by the Affordable Care Act, to prevent losses to insurers who take on sicker enrollees (Pope *et al*., 2004; McGuire *et al*., 2013; Kautter *et al*., 2014). Current risk adjustment formulas use ordinary least squares (OLS) linear regression to predict health plan payments with select demographic information and diagnosis codes from medical claims. These OLS‐based formulas are then typically evaluated with overall measures of statistical fit, such as *R* ^2^.

While *R* ^2^ is an important benchmark for evaluating global fit, it lacks information on other dimensions. As a result, risk adjustment has been criticized for not incentivizing efficient payment systems, spending, or population health management (Ash and Ellis, 2012; Layton *et al*., 2017), and for poorly estimating health costs for some groups by underpredicting their spending relative to average observed spending in the group. Underpredicting spending leads to undercompensation to the insurer, and there is evidence that insurers adjust the prescription drugs, services, and providers they cover (ie, benefit design) to make health plans less attractive for enrollees in undercompensated groups (Shepard, 2016; Carey, 2017; Geruso *et al*., 2017). Examples of undercompensated groups include enrollees with specific medical conditions, high‐cost enrollees, and partial‐year enrollees (van Kleef *et al*., 2013; Montz *et al*., 2016; Ericson *et al*., 2017). Recent research has also shown that health plan insurers have the ability to identify undercompensated groups (Jacobs and Sommers, 2015; Geruso *et al*., 2017; Rose *et al*., 2017).

What constitutes a fair or unfair algorithm depends heavily on the context. These fairness concepts and methods have been largely developed in the computer science literature (Chouldechova and Roth, 2018). We will consider risk adjustment formulas unfair if they underpredict spending for a prespecified group of enrollees, which then incentivizes differential treatment for the group via benefit design due to this undercompensation. For example, if average observed spending for individuals with mental health and substance use disorders (MHSUD) is $10 000, but average predicted spending in this group is $8000, the risk adjustment formula may be unfair for the MHSUD group by “substantially” underpredicting their spending. We define formal metrics for evaluating fairness in risk adjustment formulas using group residual errors in the next section.

Methods for addressing fairness are often divided into three categories based on the point in the learning process at which fairness is addressed: the preprocessing, fitting, or postprocessing phase. If the data are inherently biased, then preprocessing techniques are a possible solution. These methods create fair datasets by transforming or changing the data so that it is no longer biased (eg, Kamiran and Calders, 2009; Zemel *et al*., 2013). It has been shown that current spending patterns among various groups may be undesirable, and using observed spending data, we reinforce these unfair patterns. A recent study explored this concept by transferring funds to undercompensated groups in the raw data in order to promote more ideal spending patterns (Bergquist *et al*., 2019).

One of the most common fitting phase approaches in risk adjustment attempts to fix group undercompensation by adding new variables representative of the groups in the risk adjustment formula (van Kleef *et al*., 2013). While this is a straightforward idea, it can be problematic if those variables are unavailable, incentivize over‐ or underutilization of health services, or the risk adjustment formula does not recognize the improvement (Rose and McGuire, 2019). Fitting techniques in fairness include separate formulas for protected classes as well as fairness penalty terms or constraints (Kamishima *et al*., 2012; Dwork *et al*., 2018). We see intersections of these areas in the risk adjustment literature with separate formulas for enrollees with MHSUD (Shrestha *et al*., 2018) and constrained regression to reduce undercompensation for specific groups (van Kleef *et al*., 2017). Notably, separate risk adjustment formulas are already used in practice for infants and adults due to known differences in spending patterns. Nonparametric statistical machine learning methods to enhance estimation accuracy in risk adjustment have also been explored for the fitting stage (Rose, 2016; Shrestha *et al*., 2018; Park and Basu, 2018), but none of these tools are currently deployed in the US health care system.

Postprocessing techniques modify the results after fitting by, for example, creating specific classification thresholds for different groups (Hardt *et al*., 2016; Kleinberg *et al*., 2018). These methods separate fit from fairness objectives and allow using the same prediction function for multiple fairness objectives. Reinsurance, paying insurers for a portion of the costs of high‐cost enrollees, can be considered postprocessing for risk adjustment in that it reduces undercompensation for high‐risk enrollees (McGuire and van Kleef, 2018).

In this paper, we focus on the fitting phase and expand on concepts from statistics, computer science, and health economics, proposing new estimation methods and measures to improve risk adjustment formulas for undercompensated groups. We develop two new fair regression estimators for continuous outcomes that reduce residual errors for an undercompensated group by building fairness considerations directly into the objective function. We also extend a definition of fairness from the computer science and statistics literature for the risk adjustment setting while additionally considering existing measures.

Our application features the IBM MarketScan Research Databases. This set of databases contains enrollee‐level claims, demographic information, and health plan spending for a sample of individuals (and their dependents) insured by private health plans and large employers across the country. In 2014, the IBM MarketScan Research Databases were used by the federal government to develop the risk adjustment formulas for the individual health insurance Marketplaces. Thus, this data source is particularly policy relevant. The undercompensated group we focus on for this data application is enrollees with MHSUD. We select this group for two major reasons. First, individuals with MHSUD are known to have substantially undercompensated payments in current risk adjustment formulas (Montz *et al*., 2016). Second, about 20% of people in the United States have MHSUD, thus it is a priority area for policy change. Although the data are representative of only a subset of the US health insurance market, our methods are appropriate for other markets and different application settings with continuous outcomes. The methods and metrics we present are compared in this data analysis as well as simulation studies.

## 2 STATISTICAL FRAMEWORK

This section describes our approach to fair regression. It involves a suite of fairness measures for evaluating new and existing regression tools in an effort to improve risk adjustment formulas for undercompensated groups. A typical algorithmic fairness problem has an outcome *Y* and input vector ***X*** that includes a protected group A⊂X. The goal is to create an estimator for the function f(X)=Y that maps ***X*** to *Y*, while aiming to ensure that the function is fair for protected group *A*. Although our main goal is to understand whether estimation methods beyond OLS, including those we newly propose, improve fairness for risk adjustment, we also wish to focus on interpretability for stakeholders, such as government agencies, insurers, providers, and enrollees. Therefore, constrained and penalized regressions were natural choices to enforce fairness in risk adjustment for undercompensated groups.

### 2.1 Measures

The most commonly used measures of fairness are based on the notion of group fairness, striving for similarity in predicted outcomes or errors for groups. Let *g* be the set containing all $n_{g}$ enrollees with MHSUD (ie, the undercompensated group), indexed by *i*. The complement group, all $n_{c}$ enrollees without MHSUD, is denoted by $g^{c}$ and indexed by *j*. Overall sample size, $N=n_{g}+n_{c}$, is indexed by *k*. Group undercompensation is a result of large average group residuals in the risk adjustment formula. We define fairness as a function of these residual errors given that many undercompensated groups have substantially higher average health care costs. Thus, enforcing similar predicted outcomes $\hat{Y}$ between *g* and $g^{c}$ would be unfair to both. In this subsection, we present three relevant existing measures of group fairness, a new extension of fair covariance modified for group fairness with continuous outcomes, and *R* ^2^ as a metric of overall global fit.

#### 2.1.1 Mean residual difference

Comparing mean residual errors between a group *g* and its complement $g^{c}$ aims to assess fairness by evaluating whether this difference is close to zero (Calders *et al*., 2013): $1/n_{g}\sum_{i\in g}\left({\hat{Y}}_{i}-Y_{i}\right)-1/n_{c}\sum_{j\in g^{c}}\left({\hat{Y}}_{j}-Y_{j}\right)$. To date, this metric has not been applied in risk adjustment.

#### 2.1.2 Net compensation

Net compensation is a related measure from the health economics literature on the same scale as the mean residual difference (Layton *et al*., 2017): $1/n_{g}\sum_{i\in g}\left({\hat{Y}}_{i}-Y_{i}\right)$. It does not contain a term for the mean residual in the complement group. Therefore, this measure focuses on a reduction in the residuals for *g* rather than similarity in residuals between the groups. A parallel net compensation measure can be calculated for $g^{c}$.

We highlight that we intentionally take the difference ${\hat{Y}}_{i}-Y_{i}$ rather than $Y_{i}-{\hat{Y}}_{i}$ so that undercompensation for those in *g* aligns with a negative value of net compensation, in line with previous literature (eg, Bergquist *et al*., 2019). This is reflected in the mean residual difference definition above as well. We do not maintain this ordering for the corresponding estimators in Section 2.2 as we wish to penalize large undercompensation in net compensation penalized regression by *adding* to the squared error, and the squared term for mean residual difference penalized regression negates the ordering distinction.

#### 2.1.3 Predictive ratios

Predictive ratios are commonly used to quantify the underpayment for specific groups in risk adjustment (Pope *et al*., 2004): $\sum_{i\in g}{\hat{Y}}_{i}/\sum_{i\in g}Y_{i}$. Net compensation provides the absolute magnitude of the loss in dollars, whereas predictive ratios provide the relative size of the loss. Predictive ratios can also be created for $g^{c}$.

#### 2.1.4 Fair covariance

Other fairness work creates a measure based on the idea that to be fair, the predicted outcome (or residual error) and protected class must be independent. Using the covariance between the predicted outcome (or residual error) and the protected class as a proxy for independence, that work establishes a fairness measure (Zafar *et al*., 2017a; 2017b). Because this prior metric assumes that outcomes are classified into discrete categories, we extend the definition to define a new measure of fair covariance for residual errors with continuous *Y*. Our measure is given by $\text{Cov}(A,Y-\hat{Y})$, where A∈{0,1} is the random variable indicating membership in *g*. This measure is bounded by the covariance of the undercompensated group and the OLS residual, which we refer to as $c^{*}$. Our fair covariance measure allows one to see the empirical signal for systematic undercompensation through residual covariance and it can also be scaled by $c^{*}$ such that it is bounded between 0 and 1.

#### 2.1.5 Global fit

In addition to fairness measures, we also evaluate overall fit with the traditional measure used in risk adjustment, which is $R^{2}:\;1-\left\{\sum_{k}{\left(Y_{k}-{\hat{Y}}_{k}\right)}^{2}/\sum_{k}{\left(Y_{k}-{\bar{Y}}_{k}\right)}^{2}\right\}$, which we present as a percent. Given current policymaker prioritization of global metrics, it is important to compare estimators with both group and overall fit measures to understand the impact on global fit when seeking fairness for undercompensated groups.

The measures we consider above assume that the data include unbiased *Y*, which may not be the case in practice. Additionally, fairness is frequently assessed for one or two groups, as we also do here. In reality, we are often concerned about fairness for many groups. This requires the ability to define all meaningful groups, which is not always an objective task. There are also trade‐offs involved in selecting a fairness metric, and ensuring that fairness based on one definition does not necessarily guarantee a satisfying solution with respect to other fairness measures or overall fit (Kleinberg *et al*., 2016; Chouldechova, 2016; Berk *et al*., 2017). We return to these issues in our discussion. In Web Appendix A, we present a new extension of a fairness measure for comparing individual residual errors rather than mean residual errors. This *group residual difference* metric is not practical to implement at scale in risk adjustment, thus we do not deploy it here, but could be useful for small *N* settings.

### 2.2 Estimation methods

We present five methods that incorporate a fairness objective with a constraint or penalty to improve risk adjustment formulas for undercompensated groups. Two of these methods, covariance constrained regression and net compensation penalized regression, are new contributions, and all five methods will also be compared to the OLS estimator. We have a continuous spending outcome *Y*, a vector of binary health variables $H=(H_{1},\ldots ,H_{T})$, an input vector X={female,age,H}, and a coefficient vector ***θ*** indexed by *p*. For OLS, we aim to solve the following regression problem: ${\mathrm{minimize}}_{\theta }\left\{\sum_{k}{\left(Y_{k}-\sum_{p}\theta_{p}X_{kp}\right)}^{2}\right\}$.

#### 2.2.1 Average constrained regression

A previously proposed constrained regression method for risk adjustment requires that the estimated average spending for the undercompensated group is equal to the average spending, which means that net compensation for the undercompensated group is zero (van Kleef *et al*., 2017). This is achieved by including a constraint: ${\mathrm{minimize}}_{\theta }\left\{\sum_{k}{\left(Y_{k}-\sum_{p}\theta_{p}X_{kp}\right)}^{2}\right\}$, subject to $1/n_{g}\sum_{i\in g}Y_{i}=1/n_{g}\sum_{i\in g}\left(\sum_{p}\theta_{p}X_{ip}\right)$. The given constraint has been applied in the risk adjustment literature to reduce undercompensation for select groups (van Kleef *et al*., 2017; Bergquist *et al*., 2019).

#### 2.2.2 Weighted average constrained regression

The next existing method relaxes the previous constraint, allowing the estimated spending to be a weighted average of the average spending of the undercompensated group and the estimated spending under unconstrained OLS: ${\mathrm{minimize}}_{\theta }\left\{\sum_{k}{\left(Y_{k}-\sum_{p}\theta_{p}X_{kp}\right)}^{2}\right\}$, subject to $1/n_{g}\sum_{i\in g}\left(\sum_{p}\theta_{p}X_{ip}\right)=\left(1-\alpha \right)/n_{g}\sum_{i\in g}Y_{i}+\alpha /n_{g}\sum_{i\in g}\left(\sum_{p}\theta_{p}^{OLS}X_{ip}\right)$, where $\theta^{OLS}$ is the coefficient vector from the OLS. The hyperparameter α∈[0,1] is a weighting factor. When α=0, this method is equivalent to average constrained regression, and when α=1 it is equivalent to OLS. Weighted average constrained regression has been shown to reduce undercompensation for select groups in the Netherlands risk adjustment formula (van Kleef *et al*., 2017).

#### 2.2.3 Covariance constrained regression

The class of covariance methods we consider impose a constraint on the residual by requiring that the covariance between the residual and the protected class is close to zero (Zafar *et al*., 2017a; 2017b). We extend these techniques to propose a new method for our risk adjustment setting where we have a continuous residual, which has not been previously explored. In order to solve the optimization problem, we convert it into a convex problem. We simplify the covariance as follows:

$$\begin{array}{ccc} & & \text{Cov}(A,Y-\theta X)\\ & & \;=E[\{A-E(A)\}\{Y-\theta X-E(Y-\theta X)\}]\\ & & \;=E[\{A-E(A)\}(Y-\theta X)]\\ & & \;\approx \frac{1}{N}\sum_{k}\left[\left\{A_{k}-P\left(A=1\right)\right\}\left(Y_{k}-\sum_{p}\theta_{p}X_{kp}\right)\right]\\ & & \;\approx \frac{1}{N}[\left\{1-P\left(A=1\right)\right\}\sum_{i\in g}\left(Y_{i}-\sum_{p}\theta_{p}X_{ip}\right)\\ & & \;-P\left(A=1\right)\sum_{j\in g^{c}}\left(Y_{j}-\sum_{p}\theta_{p}X_{jp}\right)]. \end{array}$$

Now that we have the covariance in the form of a convex problem, we can define what we need to solve: ${\mathrm{minimize}}_{\theta }\left\{\sum_{k}{\left(Y_{k}-\sum_{p}\theta_{p}X_{kp}\right)}^{2}\right\}$, subject to $\left\{1-P\left(A=1\right)\right\}\sum_{i\in g}\left(Y_{i}-\sum_{p}\theta_{p}X_{ip}\right)-P\left(A=1\right)\sum_{j\in g^{c}}\left(Y_{j}-\sum_{p}\theta_{p}X_{jp}\right)<c$ and $\left\{1-P\left(A=1\right)\right\}\sum_{i\in g}\left(Y_{i}-\sum_{p}\theta_{p}X_{ip}\right)-P\left(A=1\right)\sum_{j\in g^{c}}\left(Y_{j}-\sum_{p}\theta_{p}X_{jp}\right)\geq -c$. Parallel to the literature for discrete categories (Zafar *et al*., 2017b), we set $c=m\times c^{*}$, where *m* is a multiplicative factor m∈[0,1] and $c^{*}$ is the covariance of the undercompensated group and the OLS residual. The upper bound for *c* occurs at m=1, which is $c^{*}$.

As we are primarily concerned with the residual of the undercompensated group being too large, we choose to instead bind the covariance on one side in our implementation of this method. In other words, we constrain the covariance to be less than some percentage of the OLS covariance (as defined by the hyperparameter *m*). A one‐sided constraint also yields faster optimization. The updated optimization problem is: ${\mathrm{minimize}}_{\theta }\left\{\sum_{k}{\left(Y_{k}-\sum_{p}\theta_{p}X_{kp}\right)}^{2}\right\}$, subject to $\left\{1-P\left(A=1\right)\right\}\sum_{i\in g}\left(Y_{i}-\sum_{p}\theta_{p}X_{ip}\right)-P\left(A=1\right)\sum_{j\in g^{c}}\left(Y_{j}-\sum_{p}\theta_{p}X_{jp}\right)<c$.

#### 2.2.4 Mean residual difference penalized regression

The relationship between penalized and constrained regressions is well recognized in statistics (Hastie *et al*., 2009), and one could equivalently reformulate the above constraints as penalties. Penalized regression has also been explored in the fairness literature. Calders *et al*. (2013) consider constrained formulations of their approaches, but propose the flexibility of penalization as an alternative due to the possibility of degenerate solutions with a high number of constraints. In their mean residual difference regression technique, one penalizes with large mean residual differences between the undercompensated group and the complement group. The coefficients minimize: $\sum_{k}{\left(Y_{k}-\sum_{p}\theta_{p}X_{kp}\right)}^{2}+\lambda {\left\{1/n_{g}\sum_{i\in g}\left(Y_{i}-\sum_{p}\theta_{p}X_{ip}\right)-1/n_{c}\sum_{j\in g^{c}}\left(Y_{j}-\sum_{p}\theta_{p}X_{jp}\right)\right\}}^{2}$, where hyperparameter λ can be user‐specified or chosen via cross‐validation, and its magnitude will be on the same scale as *Y*.

#### 2.2.5 Net compensation penalized regression

In our second new method, rather than imposing a constraint, we also formulate a penalized regression. Our regression involves the inclusion of a custom net compensation penalty term in the minimization problem: $\sum_{k}{\left(Y_{k}-\sum_{p}\theta_{p}X_{kp}\right)}^{2}+\lambda \left\{1/n_{g}\sum_{i\in g}\left(Y_{i}-\sum_{p}\theta_{p}X_{ip}\right)\right\}$. This penalty punishes estimators where the net compensation, or difference between the average spending and predicted spending for the undercompensated group, is large. We can alternatively present our new method as a constraint: ${\mathrm{minimize}}_{\theta }\left\{\sum_{k}{\left(Y_{k}-\sum_{p}\theta_{p}X_{kp}\right)}^{2}\right\}$, subject to $1/n_{g}\sum_{i\in g}\left(Y_{i}-\sum_{p}\theta_{p}X_{ip}\right)\leq z$, where the hyperparameter *z* is positive and has a one‐to‐one correspondence with, but is not equal to, λ when the constraint is binding. We choose to primarily implement this method as a penalized regression to explore differences in performance with the mean residual difference penalized regression for the same values of λ. However, simulation studies in Web Appendix B of the Supporting Information examine the performance of the constrained formulation.

### 2.3 Computational implementation

These six methods were evaluated to assess both overall fit and fairness goals with fivefold cross‐validation in our data analysis and simulations using the suite of five measures defined in Section 2.1. OLS was implemented in the R programming language with the lm() function. All other estimators were optimized using the CVXR package. This package uses disciplined convex programming to solve optimization problems and allows users to specify novel constraints and penalties (Fu *et al*., 2019).

## 3 HEALTH CARE SPENDING APPLICATION

We selected a random sample of 100 000 enrollees from the IBM MarketScan Research Databases. Age, sex, and diagnosed health conditions, all from the year 2015, were used to predict total annual expenditures in 2016. Diagnosed health conditions took the form of the established Hierarchical Condition Category (HCC) variables created for risk adjustment. HCCs were developed by the Department of Health and Human Services to group a selection of International Classification of Disease and Related Health Problems (ICD) codes into indicators for various health conditions (Pope *et al*., 2004; Kautter *et al*., 2014). We considered the 79 HCC variables currently used in Medicare Advantage risk adjustment formulas and retained the 62 HCCs that had at least 30 enrollees with the condition. See Web Appendix C for a list of the 62 HCCs included in the regression formulas. Our sample of enrollees was 52% females and between the ages of 21 and 63, with median age 45. Mean and median annual expenditures per enrollee were $6651 and $1511, respectively.

We defined enrollees with MHSUD, our protected group *A*, using Clinical Classification Software (CCS) categories. This classification system maps each MHSUD‐related ICD code to a CCS category, unlike the HCCs, which only map a subset of MHSUD‐related ICD codes. Based on CCS categories, 13.8% of the sample had a diagnosis code for MHSUD compared to 2.6% had we used HCCs. We note that we do not capture enrollees with MHSUD who do not have an ICD code for their condition(s). The mean annual expenditures for MHSUD enrollees in our sample were $11 520 versus $5880 for enrollees without MHSUD (and $3744 vs. $1274 for median annual expenditures).

**Figure 1:**
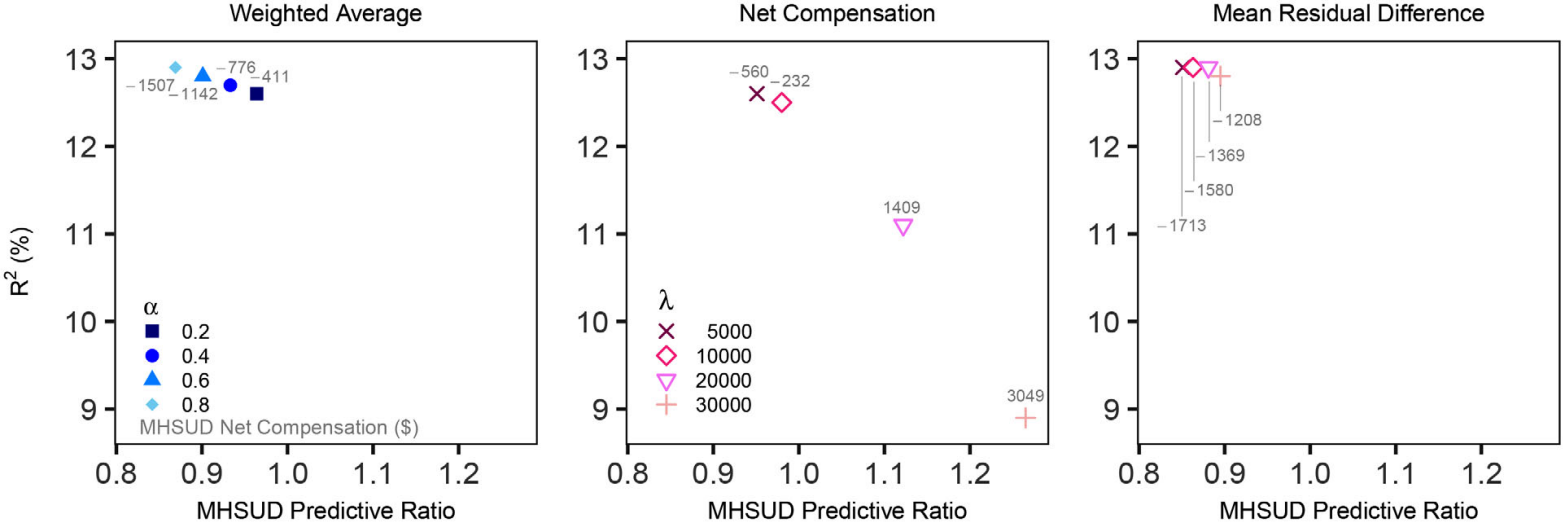
Global fit versus group fairness *Note*: Variation in cross‐validated performance by hyperparameter is plotted for three estimators. Predictive ratios for mental health and substance use disorders (MHSUD) are contrasted with overall *R* ^2^ fit. Results for all hyperparameters in the covariance constrained regression, m∈{0.2,0.4,0.6,0.8}, were extremely similar and thus omitted.

We compared each method to determine which estimators were best at reducing undercompensation for enrollees with MHSUD, and at what cost to overall statistical fit. In Table 1, we report the top estimators with respect to fairness for each of the six methods, having selected the hyperparameter value that optimizes the fairness measures (for those that have these parameters). Hyperparameter values were user‐specified from the range of plausible values. For example, in the covariance constrained regression, *m* can range from 0 to 1, and we considered m∈{0.2,0.4,0.6,0.8}. Comparisons of global fit versus group fairness for the three methods with variation in performance by hyperparameter can be found in Figure 1.

**Table 1:** Performance of constrained and penalized regression methods

		Predictive ratio		Net compensation			
Method	*R* ^2^	*g*	$g^{c}$	*g*	$g^{c}$	Mean residual difference	Fair covariance
Average	12.4%	0.996	1.001	−$46	$4	−$50	6
Covariance	12.4	0.996	1.001	−46	4	−50	6
Net compensation*	12.5	0.980	1.006	−232	34	−266	31
Weighted average†	12.6	0.964	1.011	−411	62	−473	56
Mean residual difference‡	12.8	0.895	1.032	−1208	188	−1396	164
OLS	12.9	0.837	1.050	−1872	293	−2165	256

^*^ λ=10000.

^†^ α=0.2.

^‡^ λ=30000.

*Note*: Measures calculated based on cross‐validated predicted values and sorted on net compensation. Best performing hyperparameters for each estimator (with respect to fairness measures) are displayed. Performance for covariance method was same for all *m*. $g^{c}$ is the complement of *g*.

OLS had a cross‐validated *R* ^2^ measure of 12.9%, a predictive ratio of 0.837 for individuals with MHSUD, and underestimated average MHSUD spending by −$1,872, with a mean residual difference of −$2,165. The fair covariance measure was 256. Average spending for enrollees without MHSUD was overestimated by $293 with a predictive ratio of 1.050. OLS had the worst performance along all fairness metrics while producing an *R* ^2^ only trivially higher than the competing methods.

We found the best improvement in fairness for MHSUD using the existing average constrained regression and our new covariance constrained regression. These two methods had similar performance, although not identical performance, and reduced the average undercompensation for enrollees with MHSUD to −$46 (vs. −$1,872 in the OLS), a relative improvement of 98%. They also increased the predictive ratio from 0.837 to 0.996. Enrollees without MHSUD were overestimated by only $4 and had a predictive ratio of 1.001. Both methods reduced the fair covariance measure from 256 to 6. Unsurprisingly, these two estimators were also the worst performers on overall fit as measured by *R* ^2^, although it was a loss of only 4%, from 12.9% to 12.4%. This small 0.5 percentage point loss in *R* ^2^ may be tolerable to policymakers.

Recall that the weighted average constrained regression is a compromise estimator between the OLS and average constrained regression. As α approached one in the first panel of Figure 1, the metrics more closely resembled the OLS results. As α approached zero, we saw values closer to the average constrained regression results, although α=0.2 was not only dominated by the average constrained and covariance constrained regressions, but also the net compensation penalized regression with λ=10000.

The remaining two methods were regressions with customized penalty terms to punish unfair estimates. Our proposed net compensation penalized regression varied substantially by hyperparameter (see the second panel in Figure 1), although was the third best performer overall when λ=10 000. Large λ values yielded extremely poor performance on both overall fit and fairness. At λ=20000, *R* ^2^ dropped by 12% to 11.9%, and when λ increased to 30 000, *R* ^2^ dropped to 9%, a relative reduction of 29%. These two λ values led to a large *over* *compensation* for enrollees with MHSUD. The covariance was also negative, indicating that the residual value for MHSUD was systematically too high. The mean residual difference penalized regression was less sensitive to hyperparameters compared to the net compensation penalized regression (see third panel in Figure 1). The best performance for mean residual difference penalized regression was at λ=30000; it improved on the MHSUD predictive ratio for OLS by 7% (from 0.837 to 0.895) with an *R* ^2^ loss of less than 1%. However, the best performing net compensation penalized regression had an 81% improvement over the best performing mean residual difference penalized regression when comparing MHSUD net compensation, as well as large improvements in predictive ratios (0.895 vs. 0.980) and fair covariance (164 vs. 31).

**Figure 2:**
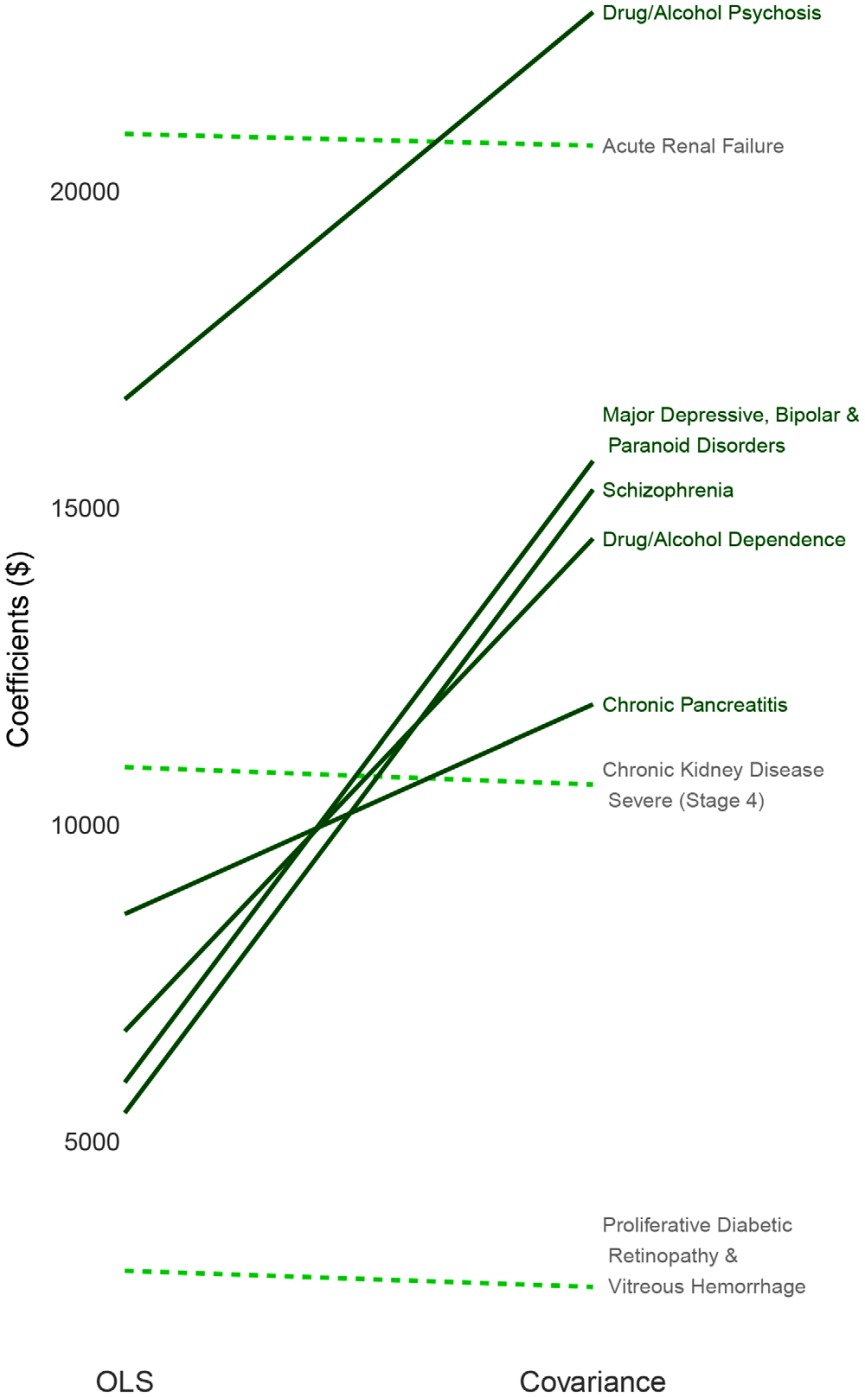
Largest coefficient changes *Note*: Increases in coefficient values from the OLS to covariance constrained regression are represented by solid lines with decreases in dashed lines. Largest five increases and largest five decreases were considered; “Chronic kidney disease, severe (Stage 4)” and “Severe hematological disorders” (both decreases) were suppressed due to large magnitudes while having small relative percentage changes of <1%.

We also examined the HCC variable coefficients for the best performing estimators, the average constrained and covariance constrained regressions, in comparison to OLS. Risk adjustment coefficients communicate incentives to insurers and providers related to prevention and care. For example, coefficients that do not reflect costs can impact an insurer's incentives in creating their plan offerings. Coefficients for the average constrained and covariance constrained regressions were nearly identical when rounded off to the nearest whole dollar, thus we display OLS versus covariance constrained regression in Figure 2. We considered the largest five increases and largest five decreases from OLS to covariance constrained regression, and observed sizable increases in the estimated coefficients associated with MHSUD. The largest relative increase was 180% for “Schizophrenia.” Relative decreases were much smaller.

## 4 SIMULATION STUDY

A set of simulation scenarios was developed to explore how these regression methods perform in other settings. We generated a population of 100 000 observations with two continuous outcomes *Y*_1_ and *Y*_2_ that were each a function of covariates in $X=(X_{1},X_{2},\ldots ,X_{9})$ and two distinct yet partially overlapping protected classes (*A*_1_ and *A*_2_) that depended on variables in ***X***. Scenario 1 considered a complex functional form for *Y*_1_ and regression estimators that were misspecified, including omitted ***X*** variables. Scenario 2 examined a less complex functional form in *Y*_2_ and regression estimators that were misspecified, including additional noise variables but no omitted ***X*** variables. A third scenario is discussed in Web Appendix B of the Supporting Information, along with complete details for the simulated population and first two scenarios. For each scenario, we drew 500 samples of N=1000 and N=10 000 observations from the simulated population of 100 000 observations. As in the data analysis, hyperparameter values were user‐specified from the range of plausible values.

**Figure 3:**
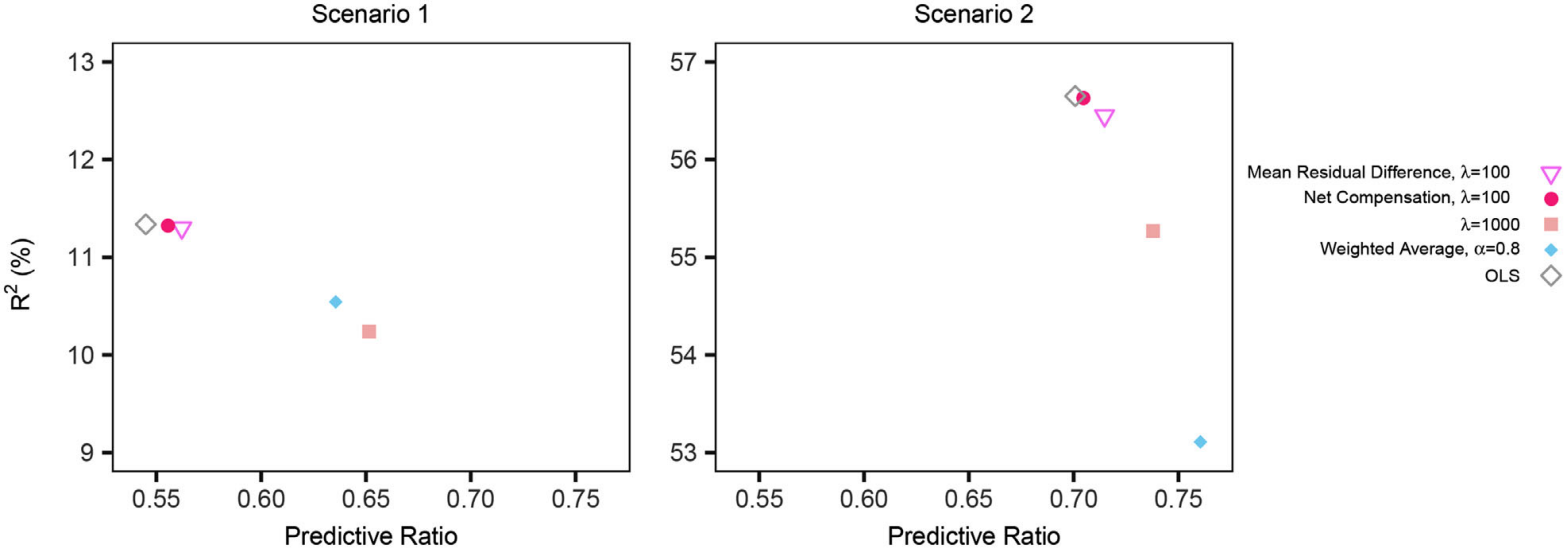
Simulation results *Note*: The plot includes OLS and estimation methods that improved fairness measures with a relative cross‐validated *R* ^2^ loss ⩽10% for N=10000. Predictive ratios for protected class *A*_1_ are contrasted with overall *R* ^2^ fit.

Selected results are presented in Figure 3, which includes OLS and those methods that improved fairness measures for protected class *A*_1_ with a relative *R* ^2^ loss ⩽10%. Notably, average constrained and covariance constrained regressions, the tied top estimators in our data analysis, do not appear. This was common across settings; average constrained and covariance constrained regressions often struggled with functional form misspecification. However, net compensation penalized regression, which performed well in our data analysis, also performed well in the simulations with respect to achieving metric balance between global fit decreases and group fit increases. Additional results are available in Web Appendix B of the Supporting Information.

## 5 DISCUSSION

We proposed new fair regression methods aiming to improve risk adjustment for undercompensated groups and asserted that a broader set of metrics is needed. As expected, there was no single method that performed the best across all the measures. One of our newly proposed techniques, net compensation penalized regression, had strong performance with respect to fairness and global fit in *both* the data analysis and simulations. Selecting the “best” method relies on subjective decisions regarding how to balance group fairness versus overall fit trade‐offs. Improvements in fairness resulted in subsequent decreases in *R* ^2^. However, for many estimators, particularly in our data analysis, improvements in fairness were larger than the subsequent decreases in overall fit. This suggests that if we allow for a slight drop in overall fit, we could greatly increase compensation for MHSUD. Policymakers need to consider whether they are willing to sacrifice small reductions in global fit for large improvements in fairness.

We used a sample of enrollees in our demonstration. At scale in a policy implementation, data from millions of enrollees would be used to estimate health spending. Solutions to group undercompensation must be scalable, and current software may or may not yet be capable of handling the sample sizes required. We tested the CVXR optimization package on larger samples and found that it was able to find solutions on a sample of 1 000 000 observations over the span of 3 days (vs. 7 h for the 100 000 enrollee sample). While the optimization results were not within the ideal optimal threshold, they still converged and the results were similar to those presented in this paper, which is promising. Future work includes additional studies regarding scalability. In our analyses, we also selected among user‐specified hyperparameter values with cross‐validation. A more thorough approach, with possibly improved results, would explore the hyperparameter space in an automated way to select values that optimize over joint fairness and fit objectives. As a general guideline, we found that λ=N/10 yielded reasonable metric balance for our newly proposed net compensation penalized regression.

We focused on one group that risk adjustment is known to disadvantage, but it is important to extend such strategies to multiple groups. Improvements for one group could result in subsequent undercompensation for other groups, and balancing fairness across an increasing number of groups is an as yet unsolved problem in risk adjustment. Our simulations examined two protected classes, and we found that improving fairness for one group did not generally help or harm the second group. Earlier research developing methods for the preprocessing phase found that reducing undercompensation for enrollees with MHSUD improved fairness measures for other groups, including enrollees with multiple chronic conditions but without MHSUD. Among the groups included in their comparisons, only enrollees with heart disease had slight reductions in fairness (Bergquist *et al*., 2019). But even the act of defining the groups poses a problem, as this can be subjective, potentially favoring larger groups with well‐funded advocacy organizations. Undercompensation could be undetected in many other lesser‐known groups. However, we can only measure undercompensation for groups that are identified by available data, and socioeconomic information, such as poverty and housing, are not available at the individual level for risk adjustment (Ellis *et al*., 2018).

Broadly, data‐driven decisions have come under scrutiny for perpetuating human biases, which certainly exists in risk adjustment. Arguments for a more comprehensive view of research results is increasing among scientific researchers today (Gibney, 2018). Recent work argues that evaluating methods from a purely statistical standpoint can lead to negative consequences, and that policy aims should be better incorporated into our research (Corbett‐Davies and Goel, 2018). Our paper follows in this spirit, and we presented additional estimators and comparisons across multiple measures for the numerous (sometimes competing) goals of risk adjustment. While we worked within the specific context of risk adjustment, the fairness methods and measures discussed here have implications for other settings with continuous outcomes, which have been understudied relative to binary outcomes.

## Supporting information

Web Appendices and Tables referenced in Sections 2–4, as well as simulated data and code in a .zip archive, are available with this paper at the Biometrics website on Wiley Online Library. Additionally, the simulated data and code are available at: github.com/zinka88/Fair‐Regression. Because the IBM MarketScan Research Databases used in this manuscript are not available for public dissemination, our repository also includes a simulated version of these data that preserves important relationships while protecting the original content, as described in the Web Appendices, along with analysis code.
