# Supplementary material for: Fair regression for health care spending
Source: Biometrics. 2020 Jan 6;76(3):973–82. doi: 10.1111/biom.13206 (PMC7540596; doi:10.1111/biom.13206)
Supplement: Supplementary file 1 — Web Appendices and Tables referenced in Sections 2–4, as well as simulated data and code in a .zip archive, are available with this paper at the Biometrics website on Wiley Online Library. Additionally, the simulated data and code are available at: github.com/zinka88/Fair‐Regression. Because the IBM MarketScan Research Databases used in this manuscript are not available for public dissemination, our repository also includes a simulated version of these data that preserves important relationships while protecting the original content, as described in the Web Appendices, along with analysis code. [file BIOM-76-973-s001.zip › biom13206-sup-0001-SuppMat.pdf]

Supporting Information for Fair Regression for Health Care Spending by

Anna Zink[1] **and Sherri Rose**[2]

[1] PhD Program in Health Policy, Harvard University, Cambridge, Massachusetts, U.S.A.

[2] Department of Health Care Policy, Harvard Medical School, Boston, Massachusetts, U.S.A.

WEB APPENDIX A: GROUP RESIDUAL DIFFERENCE

Group Residual Difference. In the fairness literature, one definition for continuous outcomes is that persons with similar Y should have similar predicted outcomes $\hat{Y}$ regardless of their protected class (Berk et al., 2017). This relies on a user-defined distance function d, such as $|Y_i - Y_j|$, to ensure that people who are 'close' have similar outcomes. We extend this definition for risk adjustment by comparing residuals rather than predicted outcomes for the two groups: $\left[1/(n_g n_c) \sum_{i \in g, j \in g^c} d(Y_i, Y_j)\{Y_i - \hat{Y}_i - (Y_j - \hat{Y}_j)\}\right]^2$. We refer to this new measure as the group residual difference. However, this measure is not practical to implement at scale in risk adjustment, which often involves millions of enrollees. The group residual difference requires comparing the residual of every enrollee in the undercompensated group to every other enrollee in the complement group. This scaling issue was also noted in the earlier work our metric extends upon (Berk et al., 2017). Our group residual difference metric can be useful in settings where N is smaller.

WEB APPENDIX B: SIMULATION STUDY DETAILS

As described in Section 4 of the main text, our simulation study population of 100,000 observations considered covariates $\boldsymbol{X} = (X_1, X_2, ..., X_9)$, two protected class indicator variables (A_1 and A_2), and two continuous outcome variables (Y_1 and Y_2), where g_1 and g_2 are the sets of observations in protected classes A_1 and A_2, respectively. X_1 was generated from a Normal distribution with mean 70 and standard deviation 15. Both X_2 and X_3 had Poisson distributions, with λ values of 10 and 35, respectively. The last six covariates ($\boldsymbol{X}_{4:9}$) were drawn from Bernoulli distributions with probabilities 0.5, 0.1, 0.05, 0.8, 0.03, and 0.2. A_1 and A_2 were also drawn from Bernoulli distributions, but depended on other generated variables in the population:

$$\begin{aligned} A_1 &\sim \text{Bernoulli}(X_4 \times X_9/2 + .01) \\ A_2 &\sim \text{Bernoulli}(X_4^2/3 + .05). \end{aligned}$$

They had prevalence rates of 6% and 22%, respectively, with 2.1% overlap. Both outcomes, Y_1 and Y_2, depended on variables in $\boldsymbol{X}$ as well as A_1 and A_2:

$$\begin{aligned} Y_1 &= (X_1 \times X_2 \times X_4) + (A_1 \times X_2 \times X_7) + (X_3 \times X_5 \times X_6) + 2^{(X_8 \times X_9)} \\ &+ (A_1 \times X_1 \times X_5) + (A_2 \times X_3 \times X_5) \\ Y_2 &= X_1 + X_2 + (X_3 \times X_4 \times X_5) + (A_1 \times X_3) + (A_1 \times A_2 \times X_1). \end{aligned}$$

We estimated regressions in three scenarios representing differing types of functional form

misspecification:

$$\begin{aligned}
\text{Scenario 1: } Y_1 &= \beta_1 X_1 + \beta_2 X_2 + \beta_3 X_3 + \beta_4 X_5 + \beta_5 X_6 + \beta_6 X_7 + \beta_7 X_8 + \beta_8 X_9 \\
\text{Scenario 2: } Y_2 &= \gamma_1 X_1 + \gamma_2 X_2 + \gamma_3 X_3 + \gamma_4 X_4 + \gamma_5 X_5 + \gamma_6 X_6 + \gamma_7 X_7 + \gamma_8 X_8 + \gamma_9 X_9 \\
\text{Scenario 3: } Y_2 &= \zeta_1 X_1 + \zeta_2 X_4 + \zeta_3 X_6 + \zeta_4 X_7 + \zeta_5 X_8 + \zeta_6 X_9.
\end{aligned}$$

Complete results for 500 draws from the population with $N = 1,000$ and $N = 10,000$ are given in Web Tables 1 and 2. Simulation data and complete analytic code to reproduce the simulation analyses are available online `github.com/zinka88/Fair-Regression`.

Web Table 1: Simulation Results, N=1,000

Scenario	Method	R^2	Predictive Ratio g_1	Net Compensation g_1	Net Compensation g_2	Fair Covariance
1	Net Compensation, $\lambda = 5000$	-2901.0%	5.96	$3231	-$273	-198.6
	Net Compensation, $\lambda = 1000$	-106.9	1.63	408	-270	-25.0
	Average	-8.8	0.98	-15	-270	0.9
	Covariance, $m = 0.2$	-8.8	0.98	-15	-270	0.9
	Mean Residual Difference, $\lambda = 5000$	-7.0	0.96	-28	-270	1.7
	Mean Residual Difference, $\lambda = 1000$	-2.0	0.89	-70	-270	4.3
	Weighted Average, $\alpha = 0.2$	-1.9	0.89	-71	-270	4.4
	Net Compensation Constraint, $z = 0.2$	0.1	0.86	-92	-270	5.7
	Weighted Average, $\alpha = 0.4$	3.5	0.80	-128	-269	7.9
	Weighted Average, $\alpha = 0.6$	7.3	0.72	-185	-269	11.4
	Mean Residual Difference, $\lambda = 100$	8.7	0.67	-215	-269	13.2
	Net Compensation, $\lambda = 100$	9.1	0.65	-227	-269	14.0
	Weighted Average, $\alpha = 0.8$	9.6	0.63	-241	-269	14.9
	Net Compensation Constraint, $z = 0.6$	9.6	0.62	-245	-269	15.1
	Net Compensation Constraint, $z = 1$	10.4	0.54	-298	-269	18.3
	OLS	10.4	0.54	-298	-269	18.4
2	Net Compensation, $\lambda = 5000$	-3436.9	2.53	217	43	-13.3
	Net Compensation, $\lambda = 1000$	-85.7	1.07	9	5	-0.6
	Average	-33.5	0.99	-1	3	0.1
	Covariance, $m = 0.2$	-33.4	0.99	-1	3	0.1
	Mean Residual Difference, $\lambda = 5000$	-26.5	0.98	-3	3	0.2
	Net Compensation Constraint, $z = 0.2$	-14.3	0.96	-6	3	0.4
	Mean Residual Difference, $\lambda = 1000$	-5.6	0.94	-8	2	0.5
	Weighted Average, $\alpha = 0.2$	-1.5	0.93	-10	2	0.6
	Net Compensation Constraint, $z = 0.6$	17.2	0.89	-16	1	1.0
	Weighted Average, $\alpha = 0.4$	23.5	0.87	-18	0	1.1
	Net Compensation Constraint, $z = 1$	39.3	0.82	-25	-1	1.5
	Weighted Average, $\alpha = 0.6$	41.3	0.82	-26	-1	1.6
	Mean Residual Difference, $\lambda = 100$	45.9	0.80	-29	-2	1.8
	Weighted Average, $\alpha = 0.8$	52.2	0.76	-34	-3	2.1
	Net Compensation, $\lambda = 100$	54.4	0.74	-37	-3	2.3
	OLS	56.0	0.70	-43	-4	2.6
3	Net Compensation, $\lambda = 5000$	-726.2	1.00	1	44	0.0
	Average	-582.7	0.97	-5	39	0.3
	Covariance, $m = 0.2$	-582.4	0.97	-5	39	0.3
	Net Compensation Constraint, $z = 0.2$	-472.3	0.94	-9	35	0.6
	Mean Residual Difference, $\lambda = 5000$	-395.8	0.91	-12	32	0.8
	Weighted Average, $\alpha = 0.2$	-358.0	0.90	-14	31	0.9
	Net Compensation Constraint, $z = 0.6$	-283.7	0.87	-18	27	1.1
	Weighted Average, $\alpha = 0.4$	-183.0	0.83	-24	22	1.5
	Net Compensation Constraint, $z = 1$	-138.0	0.81	-27	19	1.6
	Mean Residual Difference, $\lambda = 100$	-121.7	0.80	-28	18	1.7
	Weighted Average, $\alpha = 0.6$	-57.7	0.77	-33	13	2.0
	Net Compensation, $\lambda = 1000$	12.0	0.71	-42	6	2.6
	Weighted Average, $\alpha = 0.8$	18.0	0.70	-43	5	2.6
	Mean Residual Difference, $\lambda = 100$	37.5	0.66	-48	0	2.9
	Net Compensation, $\lambda = 100$	43.5	0.64	-51	-3	3.1
	OLS	44.0	0.63	-52	-4	3.2

Note: Measures are mean values across 500 samples and calculated based on cross-validated predicted values. Estimators are sorted based on net compensation and those with negative R^2 values are in shaded text.

Web Table 2: Simulation Results, N=10,000

Scenario	Method	R^2	Predictive Ratio g_1	Net Compensation g_1	Net Compensation g_2	Fair Covariance
1	Net Compensation, $\lambda = 5000$	-15.9%	1.08	$51	-$271	-3.1
	Average	-8.4	1.00	-1	-271	0.1
	Covariance, $m = 0.2$	-8.4	1.00	-1	-271	0.1
	Weighted Average, $\alpha = 0.2$	-1.3	0.90	-60	-271	3.7
	Net Compensation Constraint, $z = 0.2$	0.8	0.88	-81	-271	5.0
	Mean Residual Difference, $\lambda = 5000$	2.6	0.85	-101	-271	6.2
	Weighted Average, $\alpha = 0.4$	4.2	0.82	-120	-271	7.3
	Weighted Average, $\alpha = 0.6$	8.2	0.73	-179	-271	10.9
	Mean Residual Difference, $\lambda = 1000$	9.8	0.67	-213	-271	13.1
	Net Compensation, $\lambda = 1000$	10.2	0.65	-227	-271	13.9
	Weighted Average, $\alpha = 0.8$	10.5	0.64	-238	-271	14.6
	Net Compensation Constraint, $z = 0.6$	10.6	0.63	-241	-271	14.8
	Mean Residual Difference, $\lambda = 100$	11.3	0.56	-286	-271	17.5
	Net Compensation, $\lambda = 100$	11.3	0.56	-290	-271	17.7
	Net Compensation Constraint, $z = 1$	11.3	0.55	-297	-271	18.2
	OLS	11.3	0.55	-297	-271	18.2
2	Average	-31.2	1.00	0	3	0.0
	Covariance, $m = .2$	-31.2	1.00	0	3	0.0
	Net Compensation Constraint, $z = 0.2$	-12.2	0.97	-5	3	0.3
	Weighted Average, $\alpha = 0.2$	0.4	0.94	-9	2	0.5
	Mean Residual Difference, $\lambda = 5000$	12.3	0.91	-12	1	0.8
	Net Compensation Constraint, $z = 0.6$	18.8	0.90	-15	1	0.9
	Net Compensation, $\lambda = 5000$	22.6	0.89	-16	1	1.0
	Weighted Average, $\alpha = 0.4$	25.0	0.88	-17	0	1.1
	Net Compensation Constraint, $z = 1$	40.6	0.83	-24	-1	1.5
	Weighted Average, $\alpha = 0.6$	42.6	0.82	-26	-1	1.6
	Mean Residual Difference, $\lambda = 1000$	47.1	0.80	-29	-2	1.8
	Weighted Average, $\alpha = 0.8$	53.1	0.76	-34	-3	2.1
	Net Compensation, $\lambda = 1000$	55.3	0.74	-37	-3	2.3
	Mean Residual Difference, $\lambda = 100$	56.4	0.72	-41	-4	2.5
	Net Compensation, $\lambda = 100$	56.6	0.71	-42	-4	2.6
	OLS	56.6	0.70	-43	-4	2.6
3	Average	-637.6	1.00	-1	44	0.0
	Covariance, $m = .2$	-637.5	1.00	-1	44	0.0
	Net Compensation Constraint, $z = 0.2$	-517.1	0.96	-5	40	0.3
	Weighted Average, $\alpha = 0.2$	-392.3	0.92	-11	34	0.7
	Net Compensation Constraint, $z = 0.6$	-311.2	0.89	-15	31	0.9
	Weighted Average, $\alpha = 0.4$	-201.5	0.85	-21	25	1.3
	Net Compensation Constraint, $z = 1$	-152.2	0.83	-25	22	1.5
	Weighted Average, $\alpha = 0.6$	-65.1	0.78	-32	15	2.0
	Mean Residual Difference, $\lambda = 5000$	-28.7	0.75	-36	12	2.2
	Weighted Average, $\alpha = 0.8$	16.7	0.70	-42	5	2.6
	Net Compensation, $\lambda = 5000$	37.3	0.67	-47	1	2.9
	Mean Residual Difference, $\lambda = 1000$	38.7	0.66	-48	0	3.0
	Net Compensation, $\lambda = 1000$	43.8	0.64	-52	-3	3.2
	Mean Residual Difference, $\lambda = 100$	44.0	0.63	-52	-4	3.2
	Net Compensation, $\lambda = 100$	44.1	0.63	-53	-4	3.2
	OLS	44.1	0.63	-53	-4	3.2

Note: Measures are mean values across 500 samples and calculated based on cross-validated predicted values. Estimators are sorted based on net compensation and those with negative R^2 values are in shaded text.

WEB APPENDIX C: HIERARCHICAL CONDITION CATEGORY (HCC) VARIABLES

HCC	Description
1	HIV/AIDS
2	Septicemia, Sepsis, Systemic Inflammatory Response Syndrome/Shock
6	Opportunistic Infections
8	Metastatic Cancer and Acute Leukemia
9	Lung and Other Severe Cancers
10	Lymphoma and Other Cancers
11	Colorectal, Bladder, and Other Cancers
12	Breast, Prostate, and Other Cancers and Tumors
17	Diabetes with Acute Complications
18	Diabetes with Chronic Complications
19	Diabetes without Complication
21	Protein-Calorie Malnutrition
22	Morbid Obesity
23	Other Significant Endocrine and Metabolic Disorders
27	End-Stage Liver Disease
28	Cirrhosis of Liver
29	Chronic Hepatitis
33	Intestinal Obstruction/Perforation
34	Chronic Pancreatitis
35	Inflammatory Bowel Disease
39	Bone/Joint/Muscle Infections/Necrosis
40	Rheumatoid Arthritis and Inflammatory Connective Tissue Disease
46	Severe Hematological Disorders
47	Disorders of Immunity
48	Coagulation Defects and Other Specified Hematological Disorders
54	Drug/Alcohol Psychosis
55	Drug/Alcohol Dependence
57	Schizophrenia
58	Major Depressive, Bipolar, and Paranoid Disorders
72	Spinal Cord Disorders/Injuries
75	Myasthenia Gravis/Myoneural Disorders, Inflammatory and Toxic Neuropathy
77	Multiple Sclerosis
78	Parkinson's and Huntington's Diseases
79	Seizure Disorders and Convulsions
80	Coma, Brain Compression/Anoxic Damage
84	Cardio-Respiratory Failure and Shock
85	Congestive Heart Failure
86	Acute Myocardial Infarction
87	Unstable Angina and Other Acute Ischemic Heart Disease
88	Angina Pectoris
96	Specified Heart Arrhythmias
99	Cerebral Hemorrhage
100	Ischemic or Unspecified Stroke
103	Hemiplegia/Hemiparesis
107	Vascular Disease with Complications
108	Vascular Disease
111	Chronic Obstructive Pulmonary Disease
112	Fibrosis of Lung and Other Chronic Lung Disorders
114	Aspiration and Specified Bacterial Pneumonias
122	Proliferative Diabetic Retinopathy and Vitreous Hemorrhage
134	Dialysis Status
135	Acute Renal Failure
136	Chronic Kidney Disease, Stage 5
137	Chronic Kidney Disease, Severe (Stage 4)
161	Chronic Ulcer of Skin, Except Pressure
167	Major Head Injury
169	Vertebral Fractures without Spinal Cord Injury
170	Hip Fracture/Dislocation
173	Traumatic Amputations and Complications
176	Complications of Specified Implanted Device or Graft
186	Major Organ Transplant or Replacement Status
188	Artificial Openings for Feeding or Elimination

WEB APPENDIX D: SIMULATED ANALYSIS DATA

The IBM MarketScan Research Databases analyzed in Section 3 of the manuscript cannot be distributed online because they contain protected patient information and we were granted access via a restricted data use agreement. Thus, we created a simulated data set with similar properties using key features and relationships from the original data for reproducibility analyses of our code. The simulated analysis data described below and accompanying code to complete the analyses are available online: `github.com/zinka88/Fair-Regression`.

First, we simulated demographic variables, female and age, by sampling from a Bernoulli distribution, female $\sim$ Bernoulli(0.52), and truncated Normal distribution with lower bound a and upper bound b: age $\sim$ Normal$(44, 12, a = 21, b = 63)$. Next, we generated the 62 binary health variables $\boldsymbol{H} = (H_1, ..., H_T)$, each drawn from a Bernoulli distribution and dependent on the demographic variables female and age with coefficients determined by the relationships in the original data. To create the indicator for MHSUD, A, we generated 15 binary MHSUD CCS variables $\boldsymbol{C} = (C_1, ..., C_{15})$ dependent on age, female, and the top six HCCs correlated with MHSUD in the original data. We defined $A = 1$ for all observations with at least one MHSUD CCS; 15.7% of observations in the simulated analysis data had MHSUD compared to 13.8% in the original data.

To generate Y, we added random noise to an intermediary outcome $\ddot{Y}$ dependent on the input vector $X = \{\text{female}, \text{age}, \boldsymbol{H}, \boldsymbol{C}\}$. We note that while $\boldsymbol{C}$ was used to generate $\ddot{Y}$, it is not used later in the estimation steps as this information is not currently included in risk adjustment formulas. $\ddot{Y}$ was determined using a 2-part model. First, to capture the 10.5% of observations without spending in the original data, we generated whether any spending

occurred by creating a binary variable S with $S \sim \text{Bernoulli}(p_S)$, where $p_S = \text{logit}^{-1}[\boldsymbol{\Omega X}]$ and $\boldsymbol{\Omega}$ is a vector of coefficients based on the original data. Next, for observations with positive spending, we generated the amount of spending that occurred using a log-linear model of spending dependent on $\boldsymbol{X}$ to account for the right-skew of the spending outcome:

$$\ddot{Y} = \begin{cases} 0, & \text{if } S = 0 \\ e^{\boldsymbol{\Phi X}}, & \text{if } S = 1, \end{cases}$$

where $\boldsymbol{\Phi}$ is a vector of coefficients based on the original data. Lastly, we sampled from a truncated normal centered around each observation in $\ddot{Y}$ to add noise to the generated outcome: $Y_k \sim \text{Normal}(\ddot{Y}_k, 6000, a = 0, b = \max(Y))$, where Y_k is the predicted outcome for observation k in the simulated data. The final simulated spending outcome ranged from \$0 to \$297,206 with a mean of \$5,817 and median of \$4,881. The average spending for enrollees with MHSUD was \$6,812 versus \$5,632 for enrollees without MHSUD. R^2 under OLS was 19.7%.

The results from the simulated analysis data are shown in Web Table 3. As demonstrated in our data analysis presented in Section 3 of the main text, we likewise find that the constrained and penalized estimation methods improve fairness measures without a significant decrease in R^2. The relative rankings of all the methods are similar, although we highlight that net compensation penalized regression performs even more similarly to average constrained and covariance constrained regression methods here.

Web Table 3: Performance of Constrained and Penalized Regression Methods in Simulated Data

Method	R^2	Predictive Ratio g	Predictive Ratio g^c	Net Compensation g	Net Compensation g^c	Mean Residual Difference	Fair Covariance
Net Compensation†	18.5%	1.001	1.000	\$6	-\$1	\$7	-1
Average	18.6	0.999	1.000	-5	1	-7	1
Covariance	18.6	0.999	1.000	-5	1	-7	1
Weighted Average∓	19.0	0.984	1.004	-106	20	-127	17
Mean Residual Difference⊕	19.6	0.947	1.012	-364	68	-432	57
OLS	19.7	0.925	1.017	-512	95	-607	80

$^{\dagger}\lambda = 20000$, $^{\mp}\alpha = 0.2$, $^{\oplus}\lambda = 30000$

Note: Measures calculated based on cross-validated predicted values and sorted on net compensation. Best performing hyper-parameters for each estimator (with respect to fairness measures) are displayed. Performance for covariance method was the same for all m. g^c is the complement of g.

REFERENCES

Berk, R., Heidari, H., Jabbari, S., Joseph, M., Kearns, M., Morgenstern, J., Neel, S., and Roth, A. (2017). A convex framework for fair regression. arXiv pre-print. `arxiv.org/abs/1706.02409`.
